# CPD-CCNN: classification of pepper disease using a concatenation of convolutional neural network models

**DOI:** 10.1038/s41598-023-42843-2

**Published:** 2023-09-20

**Authors:** Yohannes Agegnehu Bezabh, Ayodeji Olalekan Salau, Biniyam Mulugeta Abuhayi, Abdela Ahmed Mussa, Aleka Melese Ayalew

**Affiliations:** 1 Department of Information Technology, University of Gondar, Gondar, Ethiopia; 2 Department of Electrical/Electronics and Computer Engineering, Afe Babalola University, Ado-Ekiti, Nigeria; 3 Department of Computer Science, University of Gondar, Gondar, Ethiopia; 4 Saveetha School of Engineering, Saveetha Institute of Medical and Technical Sciences, Chennai, India

**Keywords:** Plant sciences, Engineering

## Abstract

Agricultural products are vital to the sustainability of the economies of developing countries. Most developing countries’ economies such as Ethiopia heavily rely on agriculture. On a global scale, the pepper crop is one of the most important agricultural products in terms of human food security. However, it is susceptible to a variety of diseases which include blight leaf disease, gray leaf spot, common rust, fruit rot disease, powdery mildew symptoms on pepper leaf, and other related diseases that are all common today. Currently, more than 34 different pepper diseases have been discovered, resulting in a 33% average yield loss in pepper cultivation. Conventionally, farmers detect the disease using visual observation but this has its own demerits as it is usually not accurate and usually time consuming. In the past, a number of researchers have presented various methods for classifying pepper plant disease, especially using image processing and deep learning techniques. However, earlier studies have shown that binary classification requires improvement as some classes were more challenging to identify than others. In this study, we propose a concatenated neural network of the extracted features of VGG16 and AlexNet networks to develop a pepper disease classification model using fully connected layers. The development of the proposed concatenated CNN model includes steps such as dataset collection, image preprocessing, noise removal, segmentation, feature extraction, and classification. Finally, the proposed concatenated CNN model was evaluated, providing a training classification accuracy of 100%, validation accuracy of 97.29%, and testing accuracy of 95.82%. In general, it can be concluded from the findings of the study that the proposed concatenated model is suitable for identifying pepper leaf and fruit diseases from digital images of pepper.

## Introduction

**Figure 1:**
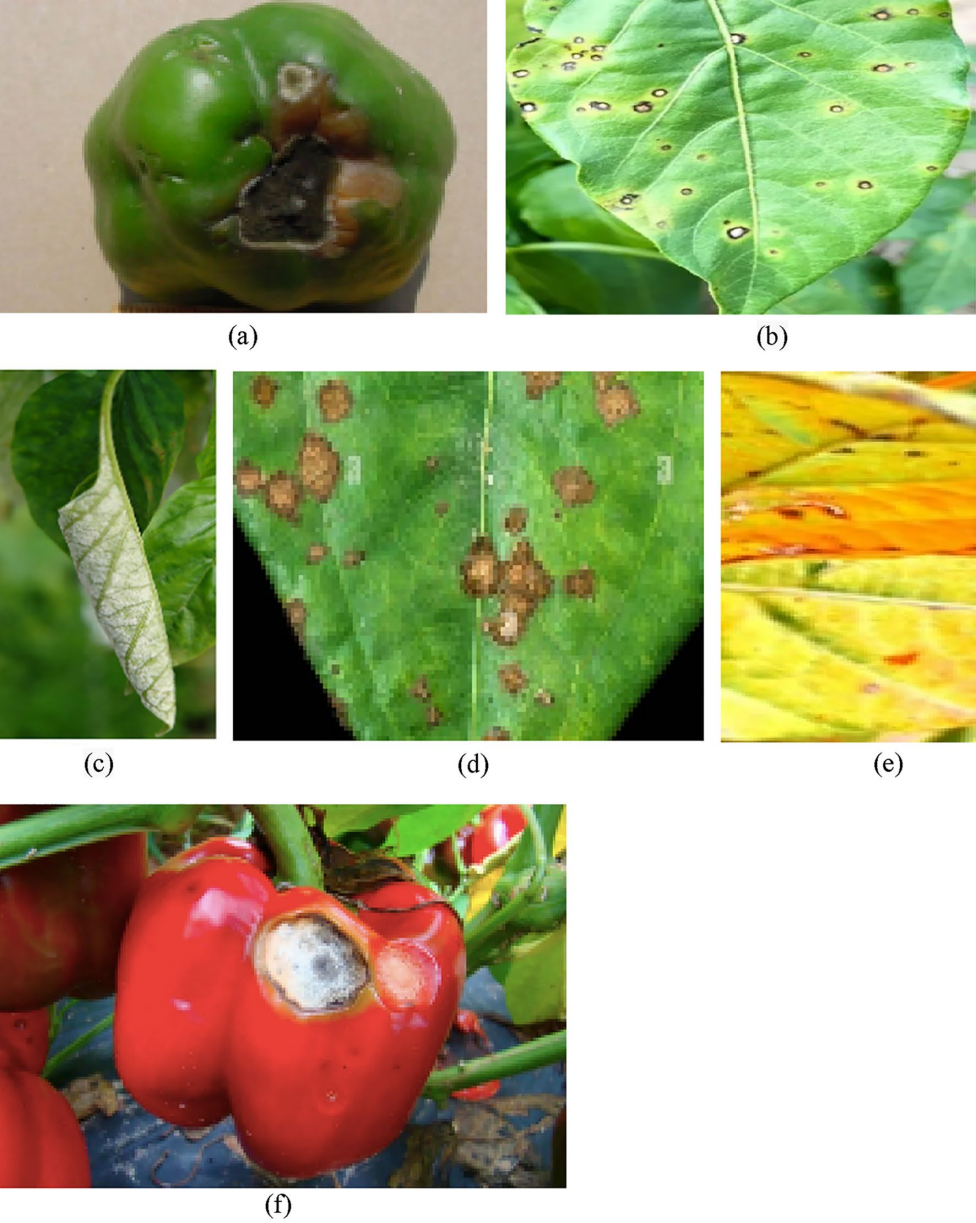
Types of pepper diseases (**a**) Blossom-end rot disease on pepper fruit, (**b**) Bacterial leaf spot on pepper plant, (**c**) Powdery mildew symptoms on pepper leaf, (**d**) Common rust of pepper, (**e**) Blight leaf disease, (**f**) Anthracnose lesion on pepper fruit.

Agriculture and its products are vital to the economies of developing countries. Plants are enormously affected by diseases which affect productivity, growth and economic well-being. Digital image processing and image analysis technologies for plant diseases are becoming increasingly important in recent times to improve and forecast agricultural yield. Pepper is a vegetable crop that is grown as a spice crop all over the world. The current study sought to ascertain the category of various peppers in Ethiopian used for market and food purposes. In Ethiopia, pepper accounts for 67.98% of all vegetable cultivated land. The Ethiopian economy is dominated by agriculture, as stated by the 2012 financial statements^1^. Pepper is an important species, fully grown in most parts of Ethiopia, and its production could play a major role in accomplishing the country’s agricultural objective of food sufficiency. Pepper diseases cause reduction in the crops growth and financial losses, as well as a reduction in the quality and quantity of agricultural products^2^. Some of the types of pepper disease are shown in Fig. 1.

This research presents the development of a concatenated classification model for pepper leaf and fruit disease classification. As a result, this research was performed to identify the various diseases of pepper to provide numerous benefits such as cost, yield, time savings, and more accurate identification. The remainder of the paper is organized based on sections as follows: Section “Related work” describes the literature review, which deals with the theoretical background, definition, and existing methods. In Section “Methodology”, a brief discussion is given on the methodology used to define the proposed model. Section “Results and discussion” presents the results and discussion of the study, and the last section describes the conclusion and features of the study.

## Related work

Numerous research efforts have been directed towards early identification of crop diseases. In this case, our research focused on pepper leaves and fruit disease classification using a concatenation of convolutional neural network (CNN) models to identify healthy pepper leaves, common rust in pepper leaves, gray-leaf spot in pepper leaves, fruit disease, and blight disease in pepper leaves.

Some studies are presented in this paper which show that a variety of techniques are being currently used for plant and leaf disease detection. The authors in^3^ evaluated the recognition of bell pepper leaf disease identification using VGG-19 on healthy and diseased pepper leaves and achieved an accuracy of 97.84%. The authors' limitation is that validation and testing accuracy were not included. According to the findings of the investigation, the authors recommended additional research for the improvements in the identification of various diseases by discovering features not only on their leaves but also on fruit diseases. One limitation of the study was the effort made to distinguish only binary bell pepper leaves from healthy and bacteria-spot disease. The authors in^4^ compared CNN architectures such as VGGNet, ResNet, and GoogLeNet classified by full-connected layers. The study's limitations include the fact that they only investigated two diseases, one of which is healthy leaf disease, and has a dataset of 1372 images, not to mention common rust, grey leaf, and fruit rot diseases. Also, they did not design their model and only tested it using pre-trained models. The authors in^5^ only examined two conditions: healthy leaves and leaf pest disease. The disadvantage was that the authors used small images that represented a dataset of 150 images for binary classification, and they also focused on only leaves with pest or disease, and the study does not mention the class which the model tested. Authors in^6^ presented a disease detection system for apple, potato, and tomato leaf using an image processing methods. The authors in^7^ addressed the problem of detecting leaf disease using the RCNN algorithm. One of this system's major flaws is the small dataset (270), for which they used binary classification rather than testing accuracy and only mentioned training accuracy. In general, most authors have used pre-trained models, machine learning algorithms, and binary classification techniques for infected or not infected pepper images.

Automatic infection detection of plant leaves and fruit diseases is critical in Ethiopian agricultural research^8^. Deep learning methods, particularly convolutional neural networks (CNN), are currently the dominant machine learning techniques for the classification of pepper leaf disease images. The authors in^9^ developed image recognition algorithms for distinguishing each part of a sweet pepper plant using the studied and applied machine learning methods for sweet pepper crop automation, and the performances of these algorithms were compared for image analysis using the normalized difference vegetation index. This feature was used in conjunction with selection metric learning methods such as K-means clustering and the morphological skeleton to classify sweet pepper parts to which the difference vegetation directory was applied. Fruit and leaf classification was performed using support vector machine (SVM) with the radial basis function kernel and the backpropagation (BP) algorithm. The accuracies of the backpropagation algorithm, the SVM, and CNN for classifying local features were 95.96%, 63.75%, and 99.50%, respectively. The authors in^10^ presented a deep learning method to detect nutrient deficiency in chilli plants and presented four classes for field detection: nitrogen deficiency (ND), potassium deficiency (PD), calcium deficiency (CD), and healthy leaves (H). The authors used a total of 270 images divided into four categories. During the investigation, they tested three types of pre-trained classifier models: MobilenetV2, RCNN Inception V2, and SSD LITE MobilenetV2. The models testing accuracies are 21.74%, 82.61%, and 4.35%, respectively. The author’s weakness was low model performance and accuracy. Authors in^11^ presented a disease identification system for the growth of sweet pepper leaves using faster R-Convolutional Neural Network (CNN) to detect plant disease from the image of leaves. In the paper, the authors used a faster R-Convolutional Neural Network (CNN) to detect plant disease of leafs in which CNN was used to determine the class of leaf diseases. They used a dataset of only 150 images to train, and the detection result is relatively good, implying that the accuracy achieved is good (97.96%), but the author's weakness is the small dataset used. The efficacy of detection using a deep learning algorithm in classifying chilli plant growth stages was presented in^12^. The authors extracted various deep learning methods used in the study, including Inception V3, ResNet50, and VGG16, and the results show that these methods performed well in terms of accuracy and stability when tested on a dataset containing 2320 images of capsicum plants at various growth stages and imaging conditions. The pre-trained models (VGG16, ResNet50, and InceptionV3) achieved accuracy rates of 91.60%, 92.01%, and 91.39%, respectively. The paper's weakness is that it only uses two types of leaf disease, infected and uninfected. The authors in^13^ were able to examine various detection methods for pepper leaf disease detection. Deep neural networks, convolutional neural networks, and recurrent neural networks (RNNs) are a few examples of detection methods used. Although the neural networks can handle noisy inputs, the algorithm's structure can be confusing. As a result, the binary detection of healthy pepper and bacterial pepper was tested using the model achieving DNN 91.386%, CNN 91.436%, and RNN 91.616%. According to the aforementioned tests, the RNN performs better, with a score of 91.616%. In^14^, the authors focused on the use of CNN to extract features and peform image recognition and detection to detect bacterial spots on bell pepper leaves that are caused by bacteria. Convolution neural networks was used to distinguish between two types of pepper leaves (healthy or bacterially infected bell pepper leaves). A test accuracy of 96.78% was achieved for the experiment. Twenty leaves were used for screening, and the procedure successfully distinguished between healthy and diseased leaves. The paper's weaknesses are only two classes were examined, while using a small dataset. The authors in^15^ presented an image processing detection system for identifying bell pepper leaf diseases. Pepper disease discovery was a method used to identify diseases from the leaves in order to address the problem. Pre-processing, segmentation, feature extraction, and detection are some of the stages used in the detection of pepper disease in the paper. The authors employed deep learning training models. The tested results for accuracy were 91.54%, 96.30%, and 96.99%, respectively, for the trained models, VGG16, Inception ResNet V2, and DenseNet120. In^16^, the authors presented a concentrated model for bacterial spot to identify bacteria-caused spot diseases. The authors validated the suggested segmentation method using classifiers such as multilayer perceptron (MLP) and SVM. The test accuracy for this experiment is 90.1% for SVM and 91.0% for MLP. In current study, we used image preprocessing, image noise removal, image segmentation, feature extraction for multi-class classification, and fruit rot disease detection, whereas other research papers used binary classification, pre-trained models, and small datasets^17^. This study presents a classification model that is a concatenation of VGG16 and AlexNet models for classification of common rust, blight leaves, grey leaf spots, fruit rot disease, healthy fruit leaf, and healthy pepper leaf.

### Contribution of the study

- *Technical contribution* This paper presents the concatenation of two neural networks (VGG16 and AlexNet) as a feature extractors to improve the learning process of acquired images of pepper. They were labeled into six classes using only binary class detection techniques, and then trained to achieve a faster and more precise model.
- *Computational time* The proposed concatenated model's computational time is designed to ensure that feature extraction of 23,127 features is more energy-efficient and takes less time than the VGG-16 and AlexNet pre-trained models with a small total number of parameters.
- *Improved performance* The proposed concatenated model improves the performance of classifying six labels of pepper leaves and pepper fruit diseases.

## Methodology

In this section, we present a detailed description of the research methodology for the development of the proposed models as well as the classification of pepper leaf and fruit diseases. A critical analysis of the methods for classification assists the authors in understanding which approach is the most suitable to conduct the research. The proposed model was used to concatenate VGG16 and AlexNet-based CNN models, and various procedures were performed such as image acquisition, dataset preparation, image preprocessing, image resizing, image segmentation, image augmentation, image feature extraction, and image classification. Authors affirm that the study complies with relevant institutional, national, and international guidelines and legislation for plant ethics.

### Architecture of the proposed model

**Figure 2:**
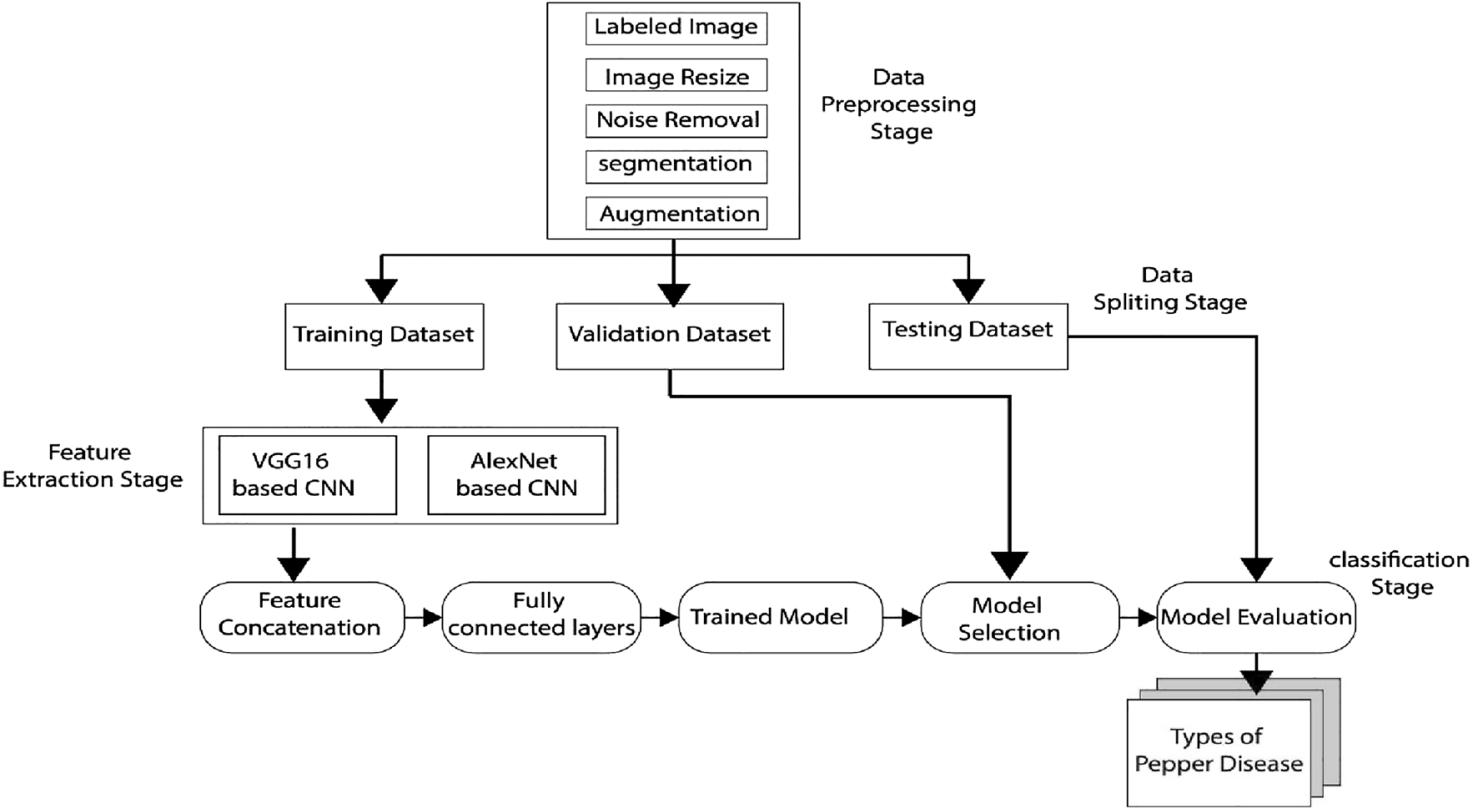
Proposed pepper leaf and fruit disease classification model.

We used digital image processing and the fully connected layers of the deep learning method to create the pepper leaf and fruit disease classification model. Deep learning was used to split the data using a variety of procedures. The dataset was divided into three parts: training, validation, and testing. For the classification model development, the dataset was divided into 70% training, 15% validating, and 15% testing. Before splitting the training, validating, and testing datasets, all of the images were resized to 128 × 128 in the first stage. The proposed models were then able to extract features that are used to classify the various pepper diseases such as common rust, blight leaves, grey leaves to spot, fruit rot disease, healthy fruit leaf, and healthy pepper leaves using a fully connected layer of the deep learning classifier. Figure 2 depicts the overall architecture of the system.

### Image dataset

The images of pepper leaves and fruit were collected from North Mecha Woreda, and the diseases are labelled by field experts at the Merawi irrigation and agriculture research institute. Images from various sources and images captured with a RGB camera are used to describe various diseases of fruit rot and pepper leaves. Each image was captured with a pixel resolution of 256 × 256 and was saved in the joint photographic group (JPG) format. The total number of images before augmentation was 1596. We duplicated the number of images using augmentation techniques because of the small number of acquired images. After duplication, the total number of images in the dataset is 3193 which improves the performance of the proposed models. We divided the datasets before training our concatenation model, and then we evaluated its performance thereafter.

### Image preprocessing

Image preprocessing is the process of rearranging dataset images before using model training. The preprocessing stage is conventionally used to improve image quality and translate images from one format to another appropriate format in order to improve the performance of models, as well as to remove noise that appears during capturing of images using the camera. In general, the goal of image preprocessing is to remove unwanted distortions from the image-assured features that are required for analysis. We used two stages in the image preprocessing stages: image resizing and background noise removal.

#### Image resizing

Before feeding the models with images, we resized the images in the dataset to reduce their size and resolution from 256 × 256 to 128 × 128. The goal of image resizing is to decrease processing time, computational complexity, and image resolution size of 256 × 256.

#### Noise removal

Image reconstruction techniques that are good in noise reduction are a serious process for background noise removal in diseases of pepper leaves and fruit. When we capture images, various noises can occur during image acquisition that reduce the performance of the model, so noise discounts are an important image processing method^18^. There are several ways to remove noise using the filtering technique in image processing, which nclude Gaussian filtering and median filtering. This study used median filtering to filter out noise and locate fruit and pepper leaf disease.

#### Image segmentation

**Figure 3:**
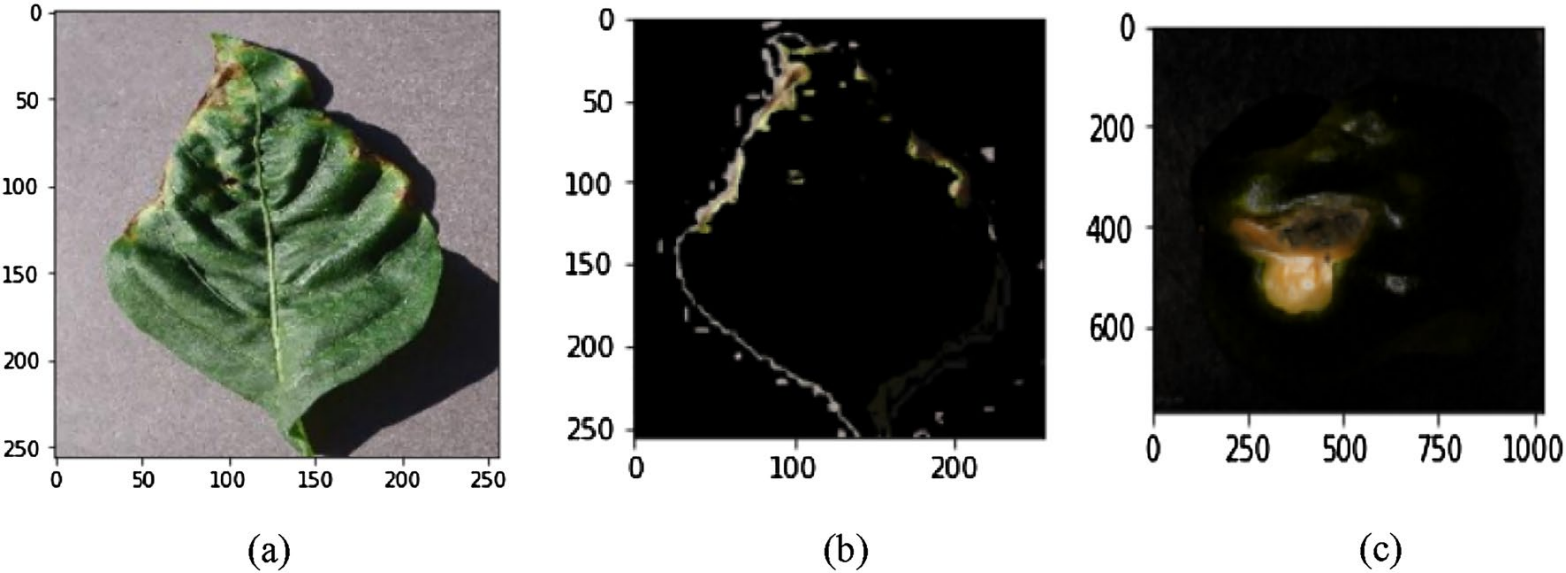
Pepper leaf and fruit (a) original image (b) segmented leaf image and (c) segmented fruit image of diseased part.

Image segmentation techniques are used to divide an image into distinct regions that contain each pixel with related features^19,20^. This represents an image feature segmentation which is more meaningful and easier to analyze^21–23^. Otsu and adaptive thresholding segmentation techniques are used as shown in Figure 3. The significant segmentation technique primarily categorizes images of common rust, blight leaves, gray leaf spot, fruit rot, healthy fruit leaf, and healthy pepper leaf disease.

#### Image augmentation

Deep learning requires a large amount of data to perform well when used to classify different types of images. Because of the inadequacy of the acquired dataset, data augmentation is used. Data augmentation is used to solve the problem of insufficient data and imbalanced image classification. Data augmentation was used to increase the size of the training set by transforming existing data. For there to be sufficient data to support the development of our model's and enhance its performance, numerous images were produced for each class using various augmentation techniques. Augmentation techniques such as the 45-degree rotation, width, and height shift range = 0.2 horizontal flipping data augmentation techniques were used.

### Feature extraction

Feature extraction is the process of extracting relevant information from the pepper's leaves and fruit and describing it in the image's nature, such as region part, edge part, and so on^24–28^. These are the characteristics that can be used to detect and classify the pepper leaves and fruit disease. We concatenated VGG16 and AlexNet to extract features because it is critical to extract deep features that help classify pepper leaves and fruit diseases. The classifier in the classification of pepper leaf and fruit disease images is a fully connected layer.

#### Feature extraction of the VGG16 and AlexNet based CNN concatenation model

The process of analyzing and extracting attributes like image color, texture, edges, and segmentation of pepper leaves and fruit rot disease is known as feature extraction. Extracted information of image features is used as the input dataset. In this paper, we concatenated VGG16 with AlexNet. The results are more accurate thanks to the developed concatenated model, which combines two models. The concatenation technique was designed to create a more accurate classification of pepper leaf and fruit diseases. After concatenating these models, we evaluated them using the fully connected layer classifier for training to validate the models.

## Results and discussion

The proposed concatenation model for automatic early classification of pepper leaf and fruit rot diseases, including common rust leaves, blight leaves, gray leaves, fruit rot, healthy leaves, and healthy fruit is examined by experimentation in this section. The concatenation of the VGG-16 and AlexNet models at the training, validation, and test phases, as well as the performance metrics that were applied, were all implemented in a systematic manner. This included the dataset preparation, simulation tools, experimental results, and performance metrics. Table 1 provides a description of the architectural design parameters of the proposed concatenated CNN model employed in this study. Six convolutions are used in the model: one max-pooling, one global average polling, one batch normalization, one dropout, and one flatten layer. To avoid overfitting, a dropout layer is added below the global average pooling and dense layer.

**Table 1:** Description of the parameters of the architecture of the proposed concatenated CNN model.

Operation	Kernel/stride	Filter size	Output shape	Parameter
2D convolution 1	1 × 1	64	32 × 32 × 96	384
2D convolution 2	1 × 1	96	32 × 32 × 16	64
2D convolution 3	3 × 3	128	32 × 32 × 64	256
2D convolution 4	1 × 1	16	32 × 32 × 128	110,720
2D convolution 5	5 × 5	32	32 × 32 × 32	12,832
2D maxpooling1	3 × 3	–	32 × 32 × 3	0
2D convolution 6	1 × 1	32	32 × 32 × 32	128
Concatenate	–	–	32 × 32 × 256	0
Batch normalization	–	–	32 × 32 × 256	1024
Global average pooling	–	–	256	0
Dropout	–	–	256	0
Flatten	–	256	256	0
Dense		524	524	134,668
Dropout	–	–	524	0
Dense	–	524	23	12,075

### Simulation environment

The experimental prototype was developed using an Anaconda environment and the Python programming language from Keras, the back-end TensorFlow, and open-source libraries are needed to implement the pepper leaves and fruit rot disease classification models. We utilized the free Google Collaborators environment in accelerated GPU option mode to train the CNN model that combines the VGG16 and AlexNet architectures. With 64 batches, the model is trained over 100 epochs.

The designed prototype was tested on a Windows 7 Hp Pavilion laptop with 64-bit operating system. The laptop is an Intel(R) Core (TM) i5 with 2.30 GHz CPU processor, with 8 GB RAM and 700 GB hard disk capacity.

### Experimental results before preprocessing of the proposed concatenated based CNN model

**Figure 4:**
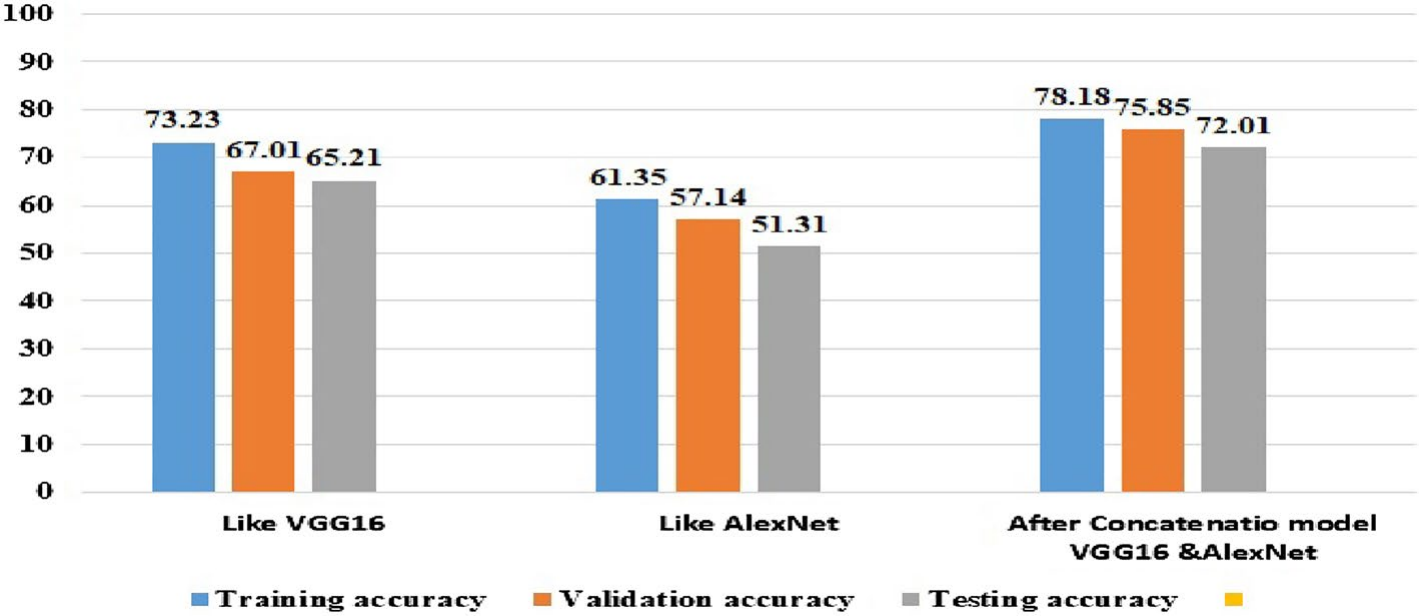
Experimental result before preprocessing of the proposed concatenated CNN model.

This was done to measure the performance of the proposed model before noise removal, segmentation of datasets, and augmentation, but the dataset size of 256 × 256 must be resized to 128 × 128 after which it is fed into the proposed model to achieve the training accuracy, validation accuracy, testing accuracy, loss, and performance of the models. As shown in Fig. 4, the performance of the proposed model is low. The reason is that the noise of images reduces the performance of the model.

### Experimental results after noise removal for the proposed concatenated based CNN model

**Figure 5:**
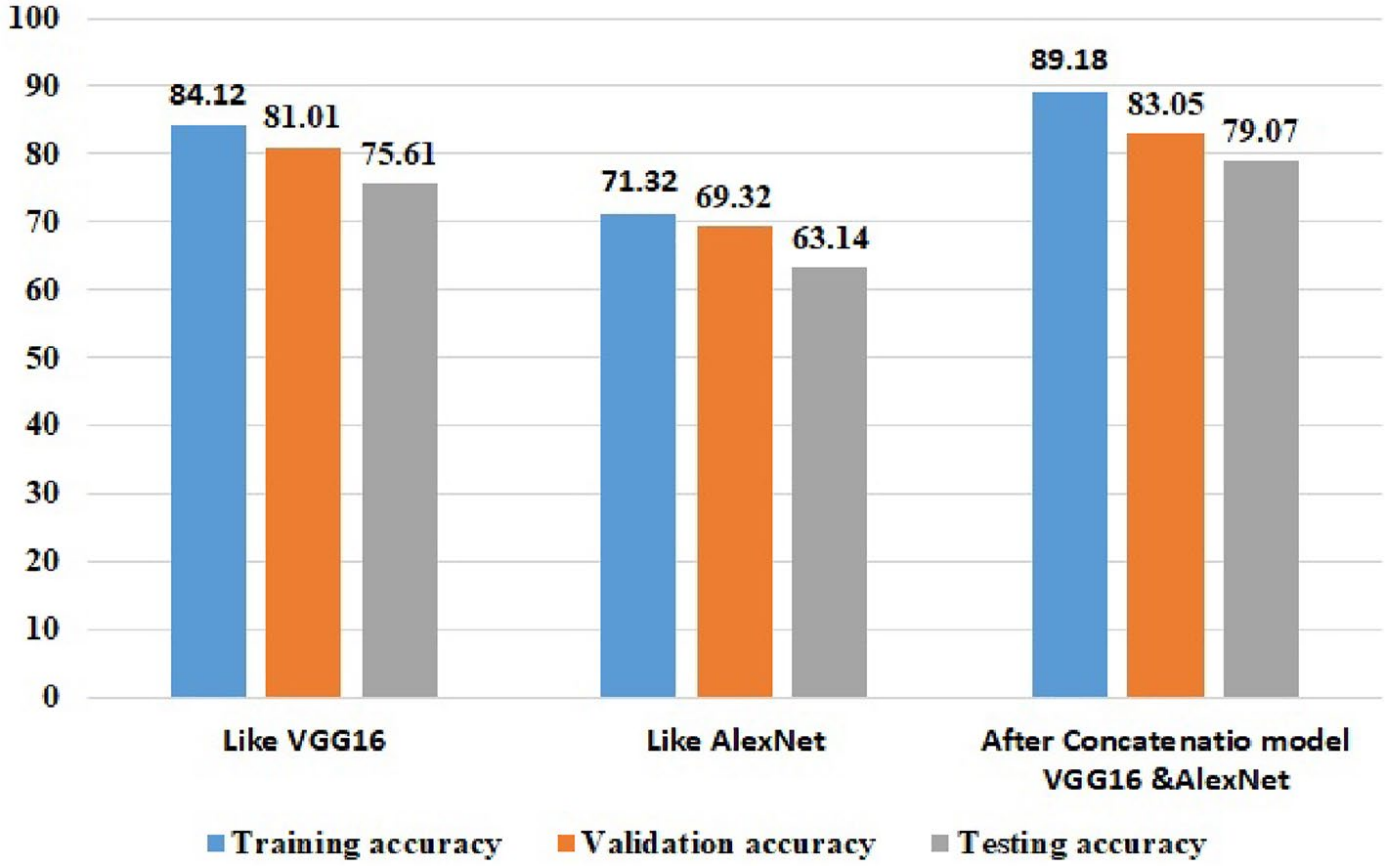
Experimental result after noise removal of the proposed concatenation based CNN model.

The filtering techniques used in the noise removal experiments are Gaussian filters. As a result, this noise reduction technique is used in the three models: VGG16, AlexNet-based CNN, and the proposed model after concatenation. When compared to the experiment one of Fig. 4, the noise removal method improve the performance of the concatenated model in the experiment. In the proposed concatenated model, noise removal was performed before image segmentation is performed. The result of the experiment is 89.18% training accuracy, 83.05% validation accuracy, and 79.07% testing accuracy, which increases some quantities as shown in Fig. 5. The concatenated models classification accuracy increases when the model is initially trained using the concatenation of two CNN-customized models: VGG-16 and AlexNet which require images of resolution, 128 × 128.

### Experimental results after segmentation of the proposed concatenated based CNN model

**Figure 6:**
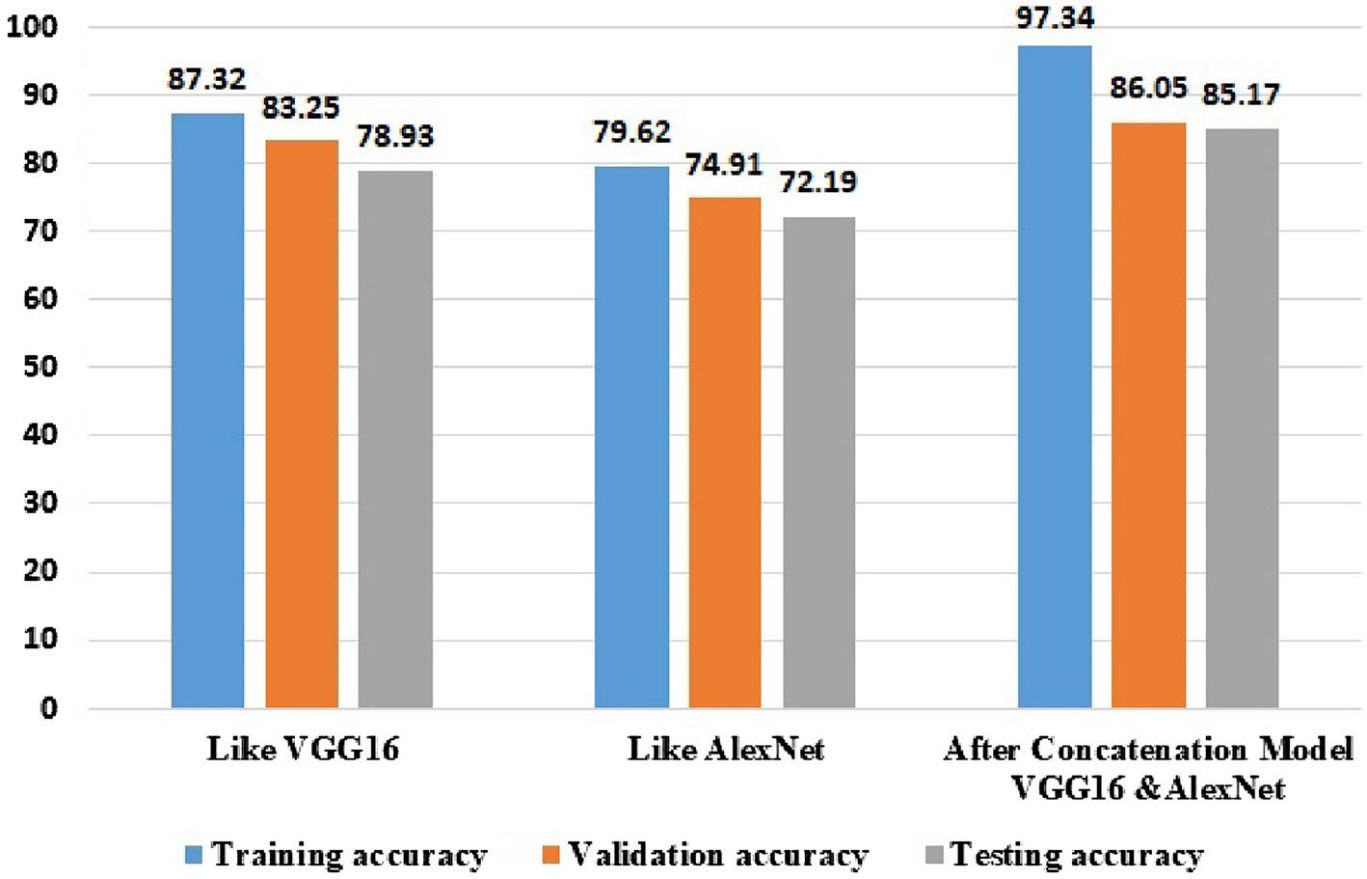
Experimental result after segmentation of the proposed concatenated based CNN model.

We used the threshold method for segmenting our image datasets under threshold segmentation. We applied adaptive thresholding, which typically takes a Gaussian filter image as input and then produces a binary image representation of the segmentation. If the pixel value is lower than the threshold, it is set to the background value; otherwise, it assumes the foreground value. After applying adaptive segmentation, we tested the experiment’s result. When compared to Otsu segmentation, the experiment’s result for Otsu segmentation testing accuracy is 83.94%, but in testing the adaptive threshold segmentation method, we achieved 85.17%. After applying adaptive segmentation to convolutional neural networks, the most common graphical curves are the training phase and validation phase curves. Figure 6 shows that without segmentation there is large overfitting between the training accuracy of 97.34%, the validation accuracy of 86.05%, and the testing accuracy of 85.17%. The training and validation loss shows the misclassification of the training and validation accuracy of the concatenated models.

#### Comparison of the proposed concatenated-based CNN model's testing performance

**Figure 7:**
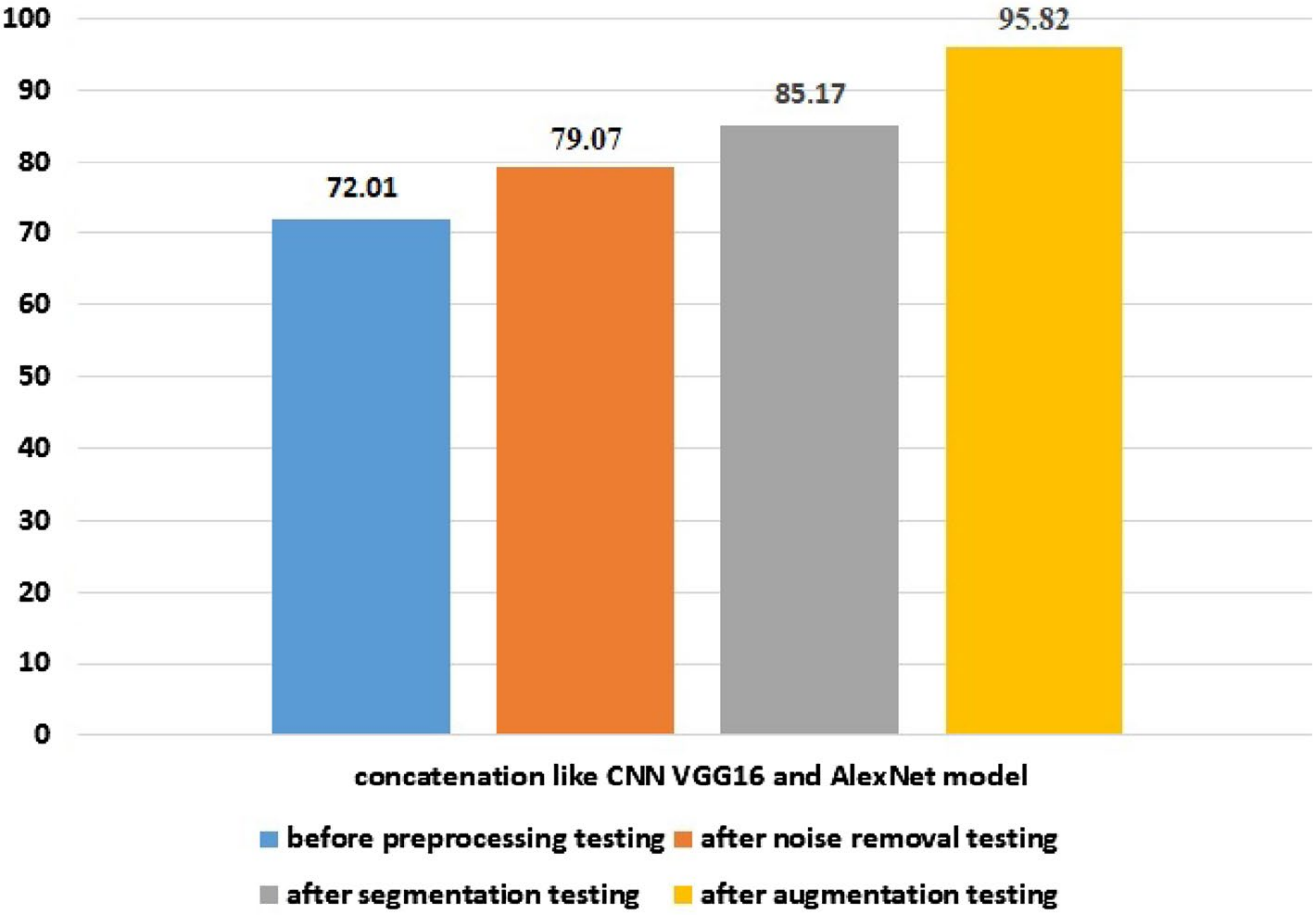
Comparison of testing performance of preprocessing of the proposed concatenated model.

We compared before preprocessing, after noise removal, after segmentation, and after dataset augmentation to determine whether the proposed model’s performance has increased. The result is described in figure format as shown in Fig. 7. Additionally, deep learning can maximize the value of their input data by preprocessing and augmenting it, which enables the best outcomes to be attained even with high-quality data. As shown in Fig. 7, the testing stage of data preprocessing is one of the basic concepts for increasing the performance of our proposed model.

#### Experimental result of training and validation accuracy curve after augmentation of the concatenated based CNN model

**Figure 8:**
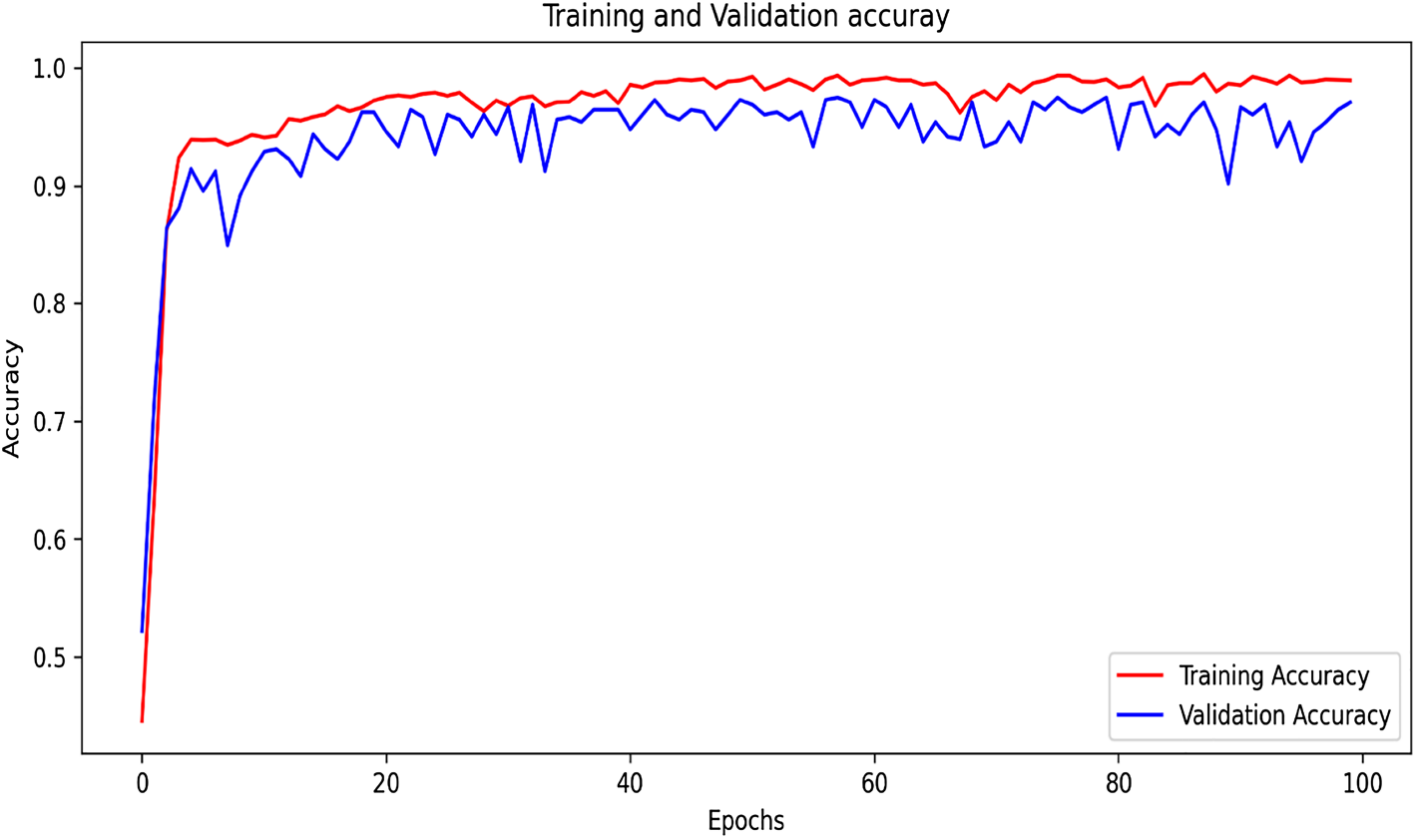
Training and validation accuracy curve after augmentation of the proposed concatenated based CNN model.

To achieve this, we concatenated by customizing feature extraction for VGG16 and AlexNet-based CNN. The proposed concatenated model is a good way of classifying the trained datasets when compared to the individual-proposed model. As shown in Fig. 8, the proposed concatenated CNN model uses mostly common graphical curves for training and its own curve for validation. In the models, the training and validation phases use 100 epochs, which greatly reduces overfitting, as shown in Fig. 8. The results show that the training accuracy is 100%, the validation accuracy is 97.29%, and the testing accuracy is 95.82%. Figure 8 shows that the model's prediction on a given input is 0.26% off in training and 9.07% off in validation.

#### Experimental result of training and validation loss curve after augmentation of the proposed concatenated based CNN model

**Figure 9:**
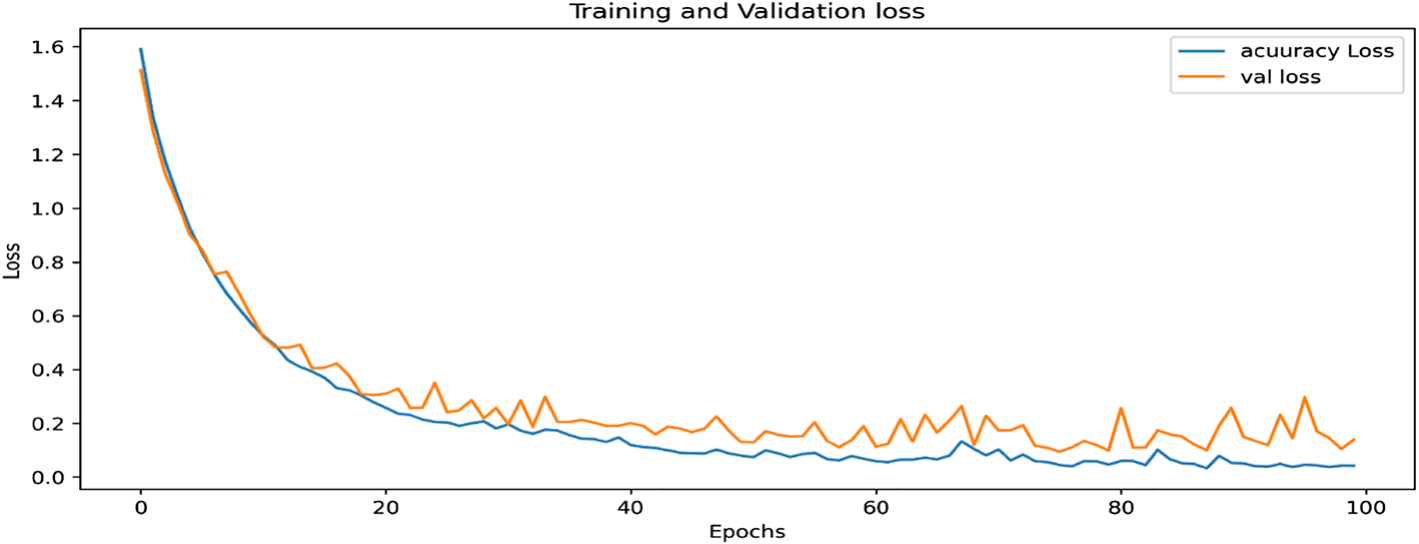
Training and validation loss curve after augmentation of the proposed concatenated CNN model.

In the concatenations, the proposed models training and validation losses shown in Fig. 9 are predicted inaccurately. Loss indicates that these models incorrectly classified true data. That is, the figure is primarily a loss curve from top to bottom. The model's prediction on a given input as inaccurately classified training accuracy loss is 0.26% and validation accuracy loss is 9.07%.

#### Performance of confusion matrix and classification result for training after augmentation of the proposed concatenated based CNN model

**Figure 10:**
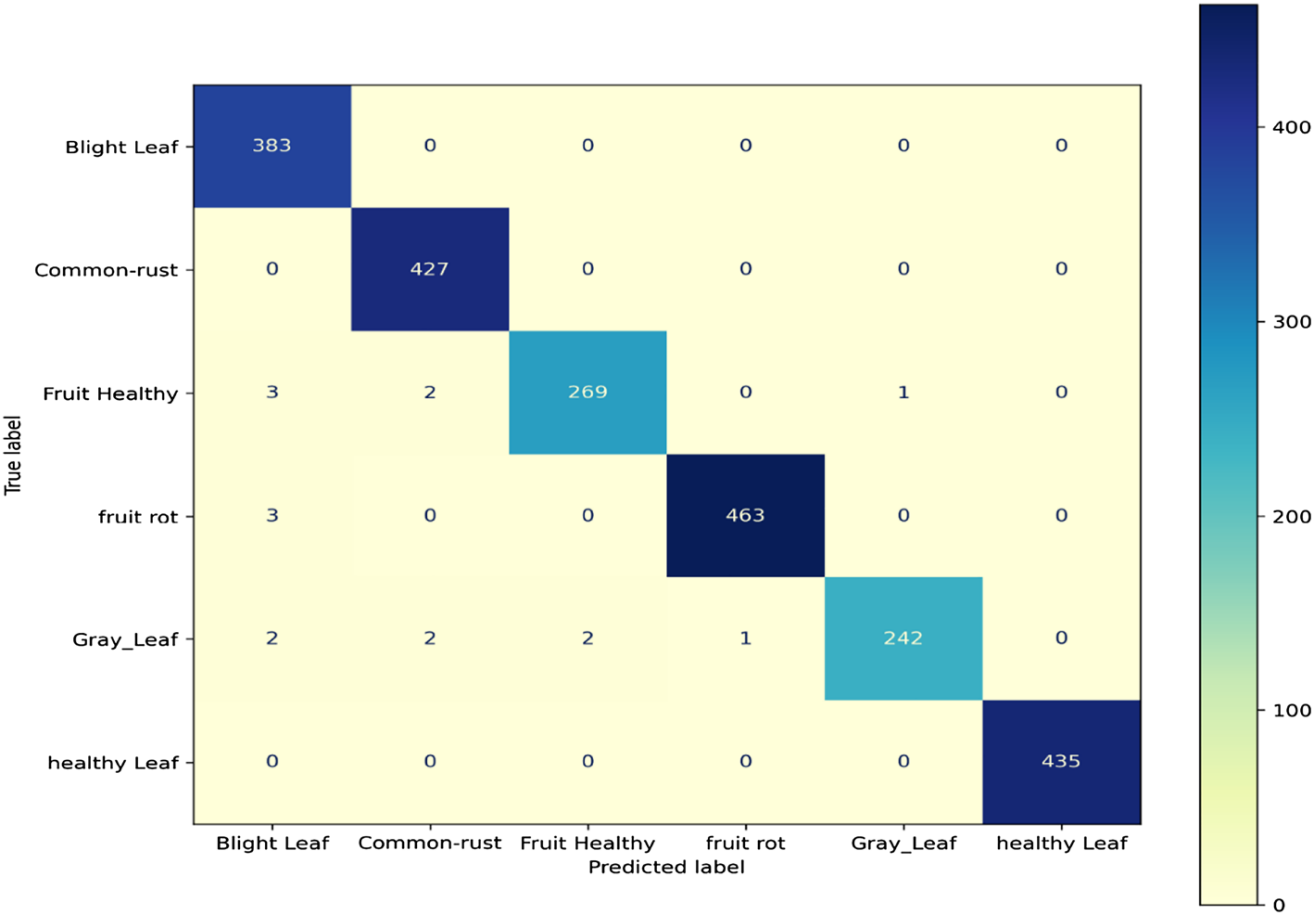
Confusion matrix of training after augmentation of the proposed concatenated CNN model.

The proposed models in the concatenations use data splits to achieve 70% training accuracy (2,235 images) across the total dataset. The training accuracy in the confusion matrix of the proposed models shows the right-to-left, vice versa, diagonal elements with the accurately classified positive rate prediction values without missing any blight images, leaving 383 out of 383 classified. That is, common rust (427 out of 427 correctly classified), healthy fruit (269 out of 274), fruit rot disease (463 out of 466), grey leaves (242 out of 249), and healthy leaves (435 out of 435) were all correctly classified. Furthermore, in the absence of a diagonal, all of the other elements shown in Fig. 10 are false predictions, implying that they are not incorrectly classified. The classification training report is a performance evaluation metric that displays the training accuracy of precision at 100%, recall at 100%, F1 score at 100%. A dataset of 2235 was used to support the training of the concatenated model, as shown in Fig. 10.

#### Performance of confusion matrix for testing after augmentation of the proposed concatenated based CNN model

**Figure 11:**
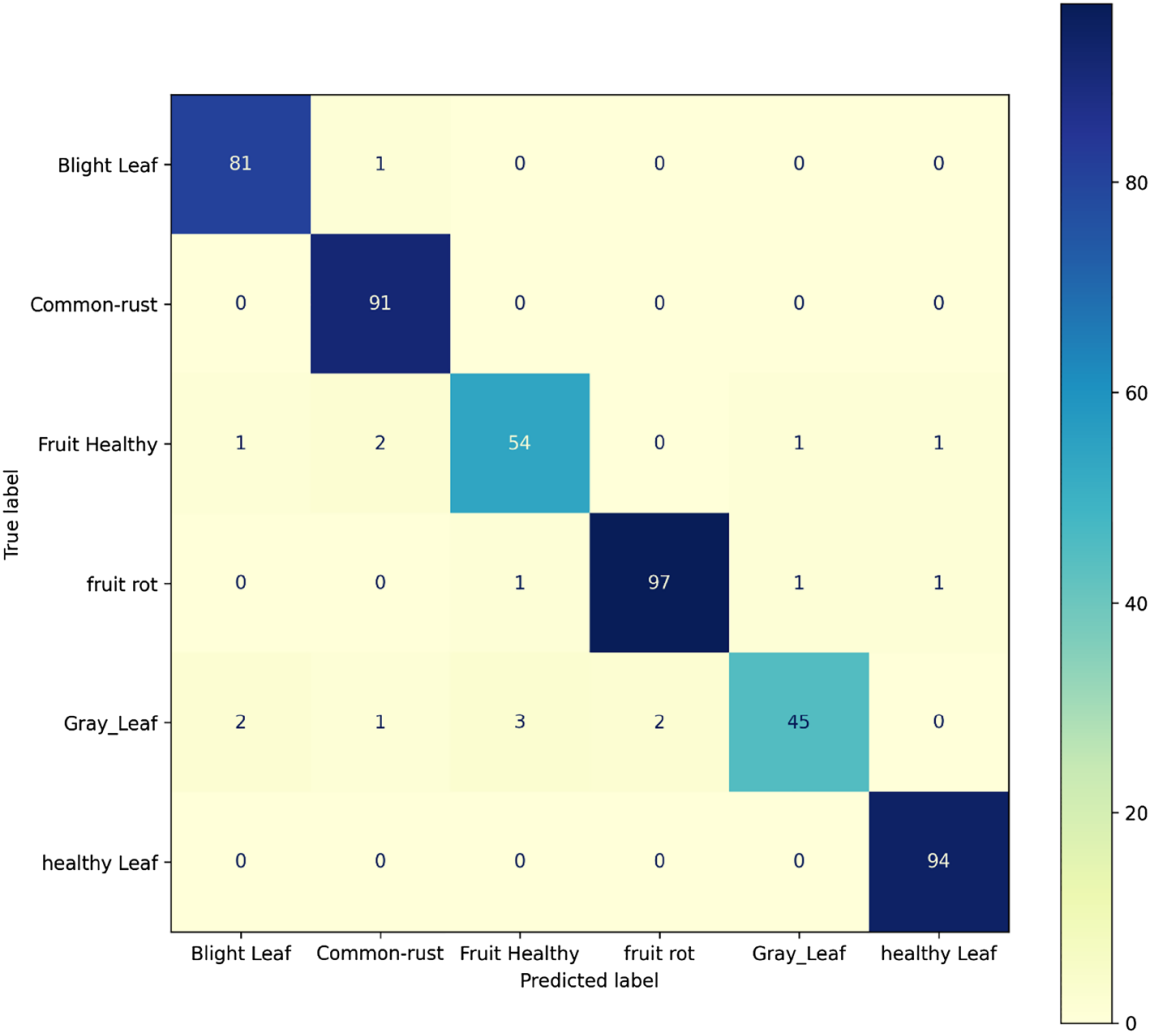
Confusion matrix of testing after augmentation of the proposed concatenated CNN model.

In the proposed concatenated model, the evaluation of the testing accuracy is a metric used after training and validation. In the confusion matrix, 15% (479 images) of the total datasets to test are made from right to left, and the diagonal elements that are positive rate accurately predicted values are displayed as 81, 91, 54, 97, 45, and 94, respectively, as shown in Fig. 11, but all other elements displayed outside of the diagonal are false rate which are inaccurately predicted. In the classification using the concatenated model, the accuracy of precision is 96%, recall is 96%, and F1 score is 96%, and the number of images in the dataset for testing is 479. Because the validation accuracy in these concatenation models is 97.28% and the testing accuracy is 95.82%, we conclude that the suggested concatenated model is superior than adapting VGG-16 and Alex Net's models individually.

In Table 2, a comparative analysis of proposed concatenated CNN model with existing systems is presented. The results show that the proposed concatenated CNN model achieves a high accuracy as compared to the number of images processed and outperforms other methods in terms of processing time which it achieved in 2 min 20 s.

**Table 2:** Comparison of proposed concatenated CNN model with existing systems.

Author	Method	Accuracy (%)	Number of images	Processing time
^14^	CNN	96.78	20	–
^15^	Deep learning models: VGG16, VGG19, ResNet50, ResNet101, ResNet152, InceptionResNetV2, DenseNet121	93.75, 92.18, 73.43, 75, 81.25, 95.31, 96.87	1596	4 min 30 s (4:30), 9:30, 7:37, 6:06, 17:37, 6:04, 3:36
^29^	CNN and Transfer learning	99.55	2478	–
^30^	CNN	96.88	2080	–
Proposed	Concatenated CNN model	95.82	3193	2:2

## Conclusion and recommendations for future work

### Conclusion

Cultivation of pepper crop is important to assure food security all over the world. In many developing countries, especially Ethiopia and Nigeria, pepper yield is greatly affected by fungus, viruses, and pest. Currently, the most prevalent pepper diseases are common leaf rust, leaf blight, gray leaves, and fruit rot, in addition to the symptoms which appear in the lower parts of leaves and fruit, which results in reduced production and loss in pepper revenue. Pepper disease can be identified conventionally by the farmer's or an expert's visual inspection. In this case, time is wasted, and many mistakes are almost certainly made, in addition to a limitation of identification tools in developing countries. In our study, those using an identification model convolutional neural network for automated classification of pepper leaves and fruit images choose to build feature extraction that concatenates for cascading VGG16, and AlexNet develops a concatenated model. In this proposed concatenation model, which was trained, validated, and tested on farmland datasets, features extracted with this proposed model were then classified using the multi-class classifier fully connected layer. We achieved this proposed model with classified experimental results of 100% training accuracy, 97.28% validation accuracy, and 95.82% testing accuracy. The results indicate that the proposed concatenated model achieves a superior performance for classified pepper leaves and fruits.

### Recommendations for future work

Based on the investigation performed and the experimental outcomes, we suggest the following recommendations for future research work:

- This study only looked at six label-class diseases of pepper leaves and fruit, but there are many other types of diseases that affect pepper not only in the leaves and fruit, but also in the stems and roots. Therefore, it is recommended that the other diseases should be investigated in future studies.
- In this research study, we used the concatenated model's fully connected layer classifier. In future works, the authors recommend the use of hybrid machine learning algorithm classifiers such as decision trees, random forests, and support vector machine to compare the performance of the model.

## Data Availability

The datasets generated during and/or analysed during the current study are not publicly available but are available from the corresponding author on reasonable request.
