# Author Correction: CPD‑CCNN: classification of pepper disease using a concatenation of convolutional neural network models

**DOI:** 10.1038/s41598-024-56022-4

**Published:** 2024-03-06

**Authors:** Yohannes Agegnehu Bezabh, Ayodeji Olalekan Salau, Biniyam Mulugeta Abuhayi, Abdela Ahmed Mussa, Aleka Melese Ayalew

**Affiliations:** 1 Department of Information Technology, University of Gondar, Gondar, Ethiopia; 2 Department of Electrical/Electronics and Computer Engineering, Afe Babalola University, Ado‑Ekiti, Nigeria; 3 Department of Computer Science, University of Gondar, Gondar, Ethiopia; 4 Saveetha School of Engineering, Saveetha Institute of Medical and Technical Sciences, Chennai, India

Correction to: *Scientific Reports* 10.1038/s41598-023-42843-2, published online 20 September 2023.

The original version of this Article contained an error in the name of Yohannes Agegnehu Bezabh, which was incorrectly given as Yohannes Agegnhu Bezabih.

The original Article has been corrected.

